# Dietary sodium sulfate supplementation improves eggshell quality, uterine ion transportation and glycosaminoglycan synthesis in laying hens

**DOI:** 10.5713/ab.24.0456

**Published:** 2024-10-28

**Authors:** Kai-bo Fu, Dong Dai, Jian-min Zhou, Jing Wang, Hai-jun Zhang, Shu-geng Wu, Guang-hai Qi, Jing Wang

**Affiliations:** 1 Key Laboratory of Feed Biotechnology of Ministry of Agriculture & Rural Affairs, Institute of Feed Research, Chinese Academy of Agricultural Sciences, Beijing, China

**Keywords:** Eggshell Quality, Glycosaminoglycan, Laying Hen, Sodium Sulfate, Uterine Ion Transportation

## Abstract

**Objective:**

This study evaluated the effects of dietary sodium sulfate (Na_2_SO_4_) supplementation on eggshell quality, uterine ion transportation, and glycosaminoglycan (GAG) synthesis.

**Methods:**

A total of 432 48-wk-old Hy-line Brown laying hens were randomly divided into 6 dietary treatments with 8 replicates of 9 birds each. The experimental laying hens were fed the corn-soybean meal diets (containing 0.15% NaCl) supplemented with 0.22%, 0.37%, 0.52%, 0.68%, 0.83%, or 0.99% Na_2_SO_4_ for 12 weeks.

**Results:**

Results showed that the eggshell breaking strength and eggshell ratio significantly increased in the 0.68% Na_2_SO_4_ group at the end of wk 56 and wk 60 (p<0.05). In addition, eggshell thickness and weight significantly increased in the 0.68% Na_2_SO_4_ group at the end of wk 60 (p<0.05). Eggshell calcium content in the 0.68% Na_2_SO_4_ group was higher than that of 0.22% and 0.99% groups (p<0.001). The concentrations of K^+^ and Ca^2+^ in the uterine fluid were significantly greater in the 0.68% group than in the other groups (p<0.05). Dietary Na_2_SO_4_ increased the gene expression of *SLC8A1*, *SCNN1A*, *ATP1B1*, and *KCNMA1* quadratically in the uterus (p<0.05), and higher values were observed in 0.68% group. Additionally, the GAG contents of the eggshell, and ATP-sulfurylase, sulfotransferase, chondroitin sulfate, and dermatan sulfate contents of the isthmus increased linearly with the increment of dietary Na_2_SO_4_ (p<0.05). There was a remarkable reduction in mammillary knob width, mammillary thickness, and the percentage of the mammillary layer (p<0.05), and an increment in mammillary knob density, effective thickness, and total thickness in the 0.68% group compared with the 0.22% and 0.99% groups (p<0.05).

**Conclusion:**

Overall, there was no dose-related difference with the increment of dietary Na_2_SO_4_ levels. The addition of 0.68% Na_2_SO_4_ in the corn-soybean basal diet (0.15% Cl) regulated uterine ion transport, increased GAG contents of eggshell, and improved eggshell ultrastructure and quality.

## INTRODUCTION

The recommended level of chloride (Cl) and sodium (Na) in the diet for laying hens is 0.15% [1], mainly provided by sodium chloride (NaCl) addition. Additionally, the use of hydrochloride additives may cause excessive Cl in the diet. When Cl intake exceeds a certain level, the quality of the eggshell might be negatively affected [2,3]. There is an increasing interest in feeding laying hens with sodium sulfate (Na_2_SO_4_) to reduce the intake of Cl and the consumption of sulfur-containing amino acids [4]. The implications of dietary Cl-free Na source replacement and addition in laying hens had been the subject of our investigations. It had been reported that the addition of dietary Na_2_SO_4_ could improve antioxidant capacity and intestinal morphology [4,5]. Nevertheless, an excessive intake of Na_2_SO_4_ in the diet could disrupt renal, hepatic, and intestinal functions, thereby affecting laying performance in poultry [4]. Various tolerance levels had been identified, including 3.0% [4] and 1.2% [6]. Previous research demonstrated that the administration of dietary supplementation containing 0.15% Cl by substituting sodium bicarbonate (NaHCO_3_) or Na_2_SO_4_ for NaCl can improve eggshell quality and laying performance [7]. In addition, it could also increase eggshell breaking strength, thickness, and the eggshell ratio. Compared with NaHCO_3_, Na_2_SO_4_ has demonstrated superior production performance [7]. Fu et al [7] and Liu et al [4] reported that feeding laying hens a diet containing 0.6% or 0.71% Na_2_SO_4_ could improve the breaking strength, thickness, and eggshell ratio of eggshells in layers. However, there has been no clear information regarding the mechanism of dietary Na_2_SO_4_ supplementation on eggshell quality.

The formation of eggshell is an ion exchange process, which requires the involvement of ions and ion transporters. It has been previously reported that uterine Na^+^, potassium (K^+^) and calcium (Ca^2+^) exchanges under the action of ion transporters to form eggshells [8,9], such as sodium channel epithelial 1 subunit *α* (*SCNN1A*), solute carrier family 8-member a1 (*SLC8A1*), ATPase Na^+^/K^+^ transporting subunit *β* 1 (*ATP1B1*), potassium calcium-activated channel subfamily m *α* 1 (*KCNMA1*). Glycosaminoglycan (GAG) is a type of proteoglycan that can regulate the ultrastructure and dominate the process of biomineralization [10–12]. Liu et al [13] reported a significant correlation between the GAG contents of eggshell membranes and breaking strength. The transit of SO_4_^2−^ is a key factor in GAG synthesis [14]. It was found that 64% of the SO_4_^2−^ used in the isthmus was synthesized into chondroitin sulfate (CS), which is a type of GAG, and that SO_4_^2−^ can regulate the content of the CS/dermatan sulfate (DS) copolymer, further improving eggshell quality [13,15]. Therefore, we hypothesized that Na_2_SO_4_ would improve eggshell quality by affecting uterine ion transportation and GAG synthesis.

The present study aimed to determine the optimal supplemental level of Na_2_SO_4_ based on the corn-soybean basal diet (containing 0.15% Cl), and the variation in eggshell mechanical quality, ultrastructure, and composition in response to dietary Na_2_SO_4_ were also observed. The possible mechanism for Na_2_SO_4_ improved eggshell quality observed in this study will provide reference data for the application of dietary Na_2_SO_4_ in the production of laying hens.

## MATERIALS AND METHODS

The experimental use of animals and related procedures were approved by the Animal Care and Use Committee of the Institute of Feed Research of the Chinese Academy of Agricultural Sciences (ACE-CAAS-20210903).

### Experimental design and diets

A total of 432 healthy Hy-line Brown laying hens at 48 wk of age were allocated into 6 groups, with 8 replicates and 9 birds per replicate. The basal diet was formulated according to the Chinese Feeding Standard of Chicken [16] and National Research Council [1] to meet the nutritional requirements (Table 1). The 6 groups of experimental laying hens were fed with the basal diets supplemented with Na_2_SO_4_ (≥95.50%) at 0.22%, 0.37%, 0.52%, 0.68%, 0.83%, and 0.99%, respectively. The Cl and Na contents of the basal diets not including NaCl and Na_2_SO_4_ were 0.06% and 0.02%, respectively. The Cl level of the basal diet was set to be 0.15% by addition of 0.15% NaCl. The total dietary Na levels of the 6 groups were 0.15%, 0.20%, 0.25%, 0.30%, 0.35%, and 0.40%. The analyzed sodium and chloride contents in each group were shown in Table 2. The diets and water were supplied ad libitum. There was no significant difference in water intake among treatments in the last trial [7], so the water intake was not reported. The feeding trial lasted for 12 wks (49 to 60 wk of age) after an acclimation period of 1 wk.

The management of laying hens was consistent with the Hy-Line guidelines. All the laying hens were fed in a fully enclosed chicken house with natural ventilation combined with longitudinal negative pressure ventilation. The temperature was controlled at 18°C to 22°C. Laying hens were housed in 3-tier battery cages and exposed to a 16 h light/8 h dark photoperiod each day. Three adjacent cages with three birds per cage (40 cm×40 cm×35 cm) were assigned as one experimental unit.

### Laying performance and eggshell quality

The number and total weight of eggs in each replicate were recorded daily during the feeding trial. The feed intake was recorded, and the egg production, average egg weight, average daily feed intake (ADFI), and feed conversion ratio (FCR) were calculated in each replicate every 2 weeks. In addition, 12 eggs were collected from each replicate for 3 consecutive days at wk 52, 56, and 60 randomly. Breaking strength was measured by an Egg Force Reader (EFR-01; Israel Orka Food Technology Ltd., Ramat Hasharon, Israel), and eggshell thickness was measured using an Egg Shell Thickness Gauge (ESTG-1; Israel Orka Food Technology Ltd.) at the equator and both poles; the values were subsequently averaged at those 3 points. The eggshell weight was measured after drying at room temperature for 48 h. The major and minor axes were measured with a Vernier caliper. The eggshell weight (%) was calculated as (eggshell weight/egg weight)×100, and the egg shape index was calculated as the major axis/minor axis.

### Minerals contents in eggshell

Four eggshells were selected randomly from each replicate as samples for measuring the Ca, phosphorus, Na, and sulfur) contents in the eggshells at the end of the trial. These eggshells were washed with distilled water to remove impurities. After drying at room temperature, the samples were mixed and ground into powder. The samples were digested by a microwave digestion instrument (MDS-10; Shanghai Xinyi Instrument Technology Ltd., Shanghai, China). Approximately 0.5 g of eggshell powder was placed in a tetrafluoroethylene digestion tank, 3 mL nitric acid and 3 mL H_2_O_2_ were added, mixed and left for 24 h. The samples were transferred to a 50 mL conical flask after digestion. The acid was removed by heating on a temperature-controlled heating plate (T<180°C), and then evaporated and concentrated to 1 to 2 mL. The liquid in the conical flask was transferred to a 25 mL volumetric flask and set aside. The Ca and Na contents were analyzed via atomic absorption spectroscopy (Zeenit700 P; Analytik Jena Ltd., Jena, Germany), the phosphorus content was analyzed via spectrophotometry (UV-2000; Shimadzu Ltd., Kyoto Japan), and the sulfur content was analyzed via inductively coupled plasma-mass spectrometry (Agilent 7700x; Agilent Technology Ltd., Beijing, China) [17,18].

### Ion concentrations and pH values in serum and uterine fluid

At 60 wks of age, two hens were selected from each replicate (same as the hens were selected in Laying performance and Eggshell quality), one hen was housed in one cage, and the laying time was recorded for 7 consecutive days. We selected and euthanised hens at 18.5 h post oviposition to collect serum and uterine fluid samples after the trial. The Na^+^, Cl^−^, K^+^, Ca^2+^, and HCO_3_^−^ concentrations and pH values in the serum and uterine fluid were determined instantly using an automatic blood analyzer (PL2000; Perlong Medical Ltd., Jiangsu, China).

### RNA isolation and real-time polymerase chain reaction

Two hens were selected and euthanized from each replicate at 18.5 h post oviposition to collect uterus randomly. Before RNA isolation, the thawed samples were ground in liquid nitrogen. Total RNA was extracted from tissues using TRIzol reagent (DP419; Tiangen Biotech Ltd., Beijing, China) according to the manufacturer’s instructions, and RNA purity and concentration were determined using a spectrophotometer (ND5000; Thermo Fisher Scientific Ltd., Portland, OR, USA). The integrity of the ribosomal RNA bands was confirmed on agarose gels. The quantification of mRNA consisted of a two-step reaction of reverse transcription and polymerase chain reaction (PCR). Reverse transcription was performed using 2 μg of RNA and a FastQuant RT Kit (KR106; Tiangen Biotech Co. Ltd., Portland, OR, USA). Real-time quantitative PCR was carried out with a CFX96 Touch Real-time PCR detection system (Bio-Rad Laboratories, Inc., Hercules, CA, USA) and SuperReal PreMix Plus (SYBR Green, FP205, Tiangen Ltd.). The PCR cycling program was as follows: initial denaturation for 15 min at 95°C, followed by 40 cycles of 95°C for 10 s, annealing and extension for 30 s at 60°C, each sample with 3 replicates. The results were normalized to that of *β-actin*, and the relative gene expression levels were calculated via the 2^−ΔΔCT^ method by Livak and Schmittgen [19]. The primer sequences for *SCNN1A*, *SLC8A1*, *ATP1B1*, *KCNMA1*, and *β-actin* are given in Table 3. Sequences based on Jonchère [20].

### Sulfated glycosaminoglycan in eggshell

At the end of wk 60, 4 eggshells from each replicate were immersed in 5% ethylenediaminetetraacetic acid (EDTA) to separate the calcified eggshell from the membrane and mixed as a sample. Approximately 1 g of calcified eggshell was used to measure the content of sulfated GAG. GAG content was determined using the methods of Ha et al [11] and Xiao et al [21].

### Determination of ATP-S, SULT, CS, and DS in the isthmus portion

Two hens were selected and euthanised at 9 h post oviposition to collect the isthmus portion randomly. The contents of ATP-sulfurylase (ATP-S) and sulfotransferase (SULT) were determined by a chicken enzyme-linked immunoassay (ELISA) kit (Shanghai Enzyme-linked Biological Technology Ltd., Shanghai, China). The contents of CS and DS were determined by chicken ELISA kits (Shanghai Xin Yu Biotech Ltd., Shanghai, China).

### Eggshell ultrastructure

We collect eggshell samples at 18.5 h post oviposition from 0.22%, 0.68%, or 0.99% Na_2_SO_4_ groups for eggshell ultrastructure observation. Four eggshell samples were collected from each replicate randomly, then two pieces of eggshell approximately 0.5 to 1 cm^2^ in length were selected from the equatorial section of each eggshell sample, a total of 8 pieces from each replicate were selected. Both the inside and outside of the eggshell were cleaned with distilled water to remove albumen and dirt, dried without affecting the vertical profile of the eggshell, fixed in a copper block with conductive glue, and sprayed with gold powder. To calculate the mammillary knob density, the sample was soaked in 1.0 N sodium hydroxide for 72 h to remove the albumen before being washed with the method of Gongruttananun [22]. The samples were imaged by scanning electronic microscopy (FEI Quanta 600; Thermo Fisher Scientific Ltd.). The effective thickness (covering cuticle, vertical crystal layer, and palisade layer), mammillary thickness, mammillary knob width, and total thickness were determined and defined as described by Zhang et al [23]. All the standard error of the mean (SEM) images were taken at 200× magnification.

### Statistical analysis

Normality was assessed before the data analysis of the egg production rate was performed. All the data were analyzed using one-way analysis of variance (ANOVA), and the means were compared using Duncan’s multiple range test in SAS (SAS Institute Inc., Cary, NC, USA). The linear and quadratic effects of the supplemental Na_2_SO_4_ concentration were assessed using regression analysis. Differences were considered statistically significant at p≤0.05. The data are presented as the mean and pooled SEM.

The regression model was as follows:


$$\text{Yij}=\alpha +\beta 1\text{Xi}+\text{eij }(\text{linear regression})\;\text{and Yij }\alpha +\beta 1{\text{Xi}}^{2}+\text{eij }(\text{quadratic regression}).$$

Yij was the response variable, α was the intercept, β1 and β2 were regression coefficients, Xi was the studied factor effect that included Na_2_SO_4_ (i = 0.22%, 0.37%, 0.52%, 0.68%, 0.83%, and 0.99%), and eij was the observational error for (ij)th observation.

## RESULTS

### Laying performance

No mortality was observed during the trial. No significant differences were observed in laying performance by various supplemental Na_2_SO_4_ concentrations. As shown in Table 4, there were no significant differences in average egg weight, egg production, ADFI, or FCR among the groups (p>0.05).

### Eggshell quality

The eggshell quality was improved by 0.52% and 0.68% supplementation with Na_2_SO_4_ (Table 5). The eggshell quality improved notably in the 0.68% Na_2_SO_4_ group. The breaking strength was greater in the 0.68% Na_2_SO_4_ group than in the other groups (p<0.05). Similarly, compared with those in the 0.22% and 0.37% Na_2_SO_4_ groups, the eggshell ratio in the 0.68% Na_2_SO_4_ group increased at the end of wk 56 (p<0.05). At the end of wk 60, the breaking strength, eggshell thickness, and eggshell weight increased in the 0.52% and 0.68% Na_2_SO_4_ groups compared with those in the 0.22%, 0.83%, and 0.99% groups, respectively (p<0.05). There was no significant difference in the egg shape index among the experimental groups (p>0.05).

### Minerals contents in the eggshell

In this study, the 0.68% Na_2_SO_4_ group had a greater Ca concentration than the other groups (Table 6). There was a quadratic response in the Ca content among the different treatments. In contrast to that in the 0.22% and 0.99% Na_2_SO_4_ groups, a higher concentration of Ca was observed in the 0.68% group (p<0.001). Dietary Na_2_SO_4_ increased eggshell Na content linearly (p<0.05).

### Serum and uterine fluid ion concentrations

Changes in the ion concentrations in the serum and uterine fluid induced by dietary Na_2_SO_4_ were found in this study (Table 7). In particular, the changes in Ca^2+^ concentration in uterine fluid were in accordance with the changes in eggshell Ca content. With increasing Na_2_SO_4_ supplementation, the serum Na^+^ concentration significantly increased (p<0.001). The Ca^2+^ concentration increased linearly, while the K^+^ concentration decreased linearly (p<0.05). The Na^+^ concentrations in the 0.83% and 0.99% Na_2_SO_4_ groups were greater than those in the 0.22% and 0.37% Na_2_SO_4_ groups (p<0.05). For uterine fluid, the K^+^ concentrations in the 0.83% and 0.99% Na_2_SO_4_ groups were significantly increased, while the Na^+^ concentrations in the 0.83% and 0.99% Na_2_SO_4_ groups were significantly decreased (p<0.05). The Ca^2+^ concentration was markedly greater in the 0.68% Na_2_SO_4_ group than in the other groups (p<0.05).

### Expression of ion transfer and transporter genes in the uterus

**Figure 1:**
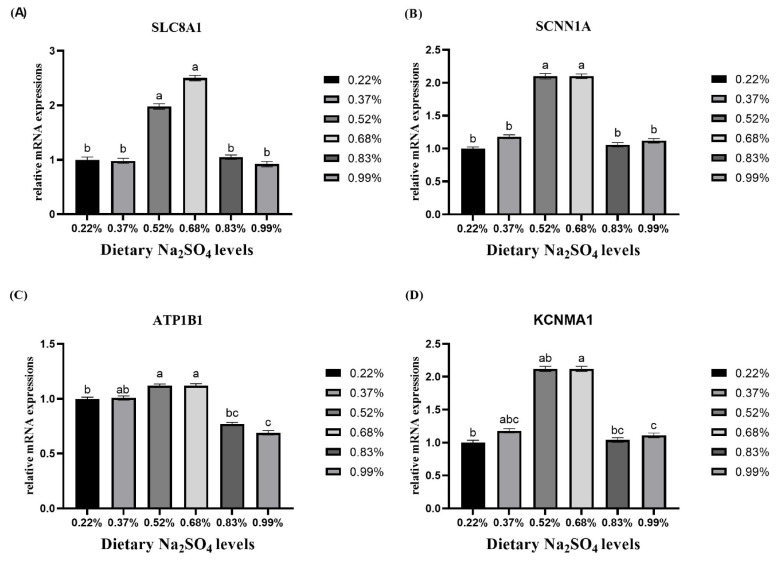
Effect of dietary sodium sulfate (Na_2_SO_4_) supplementation on uterus relative mRNA expression of ion transporters at 18.5 h post oviposition of layers. The values are the means of 8 replicates with 2 birds each. ^a–c^ The different superscript letters denote significant differences among experimental treatments. *SLC8A1*, solute carrier family 8-member a1; *SCNN1A*, sodium channel epithelial 1 subunit *α*; *ATP1B1*, ATPase Na^+^/K^+^ transporting subunit *β*1; *KCNMA1*, potassium calcium-activated channel subfamily m *α*1.

A greater expression was found in the 0.68% Na_2_SO_4_ group than in the other groups, which indicated that ion transport could be promoted by this level (Figure 1). In this study, *SLC8A1*, *SCNN1A*, and *ATP1B1* expression in the 0.52% and 0.68% Na_2_SO_4_ groups was greater than that in the other groups (p<0.05). *KCNMA1* expression in the 0.68% Na_2_SO_4_ group was significantly greater than that in the other groups (p<0.05).

### The regulation of glycosaminoglycan

**Figure 2:**
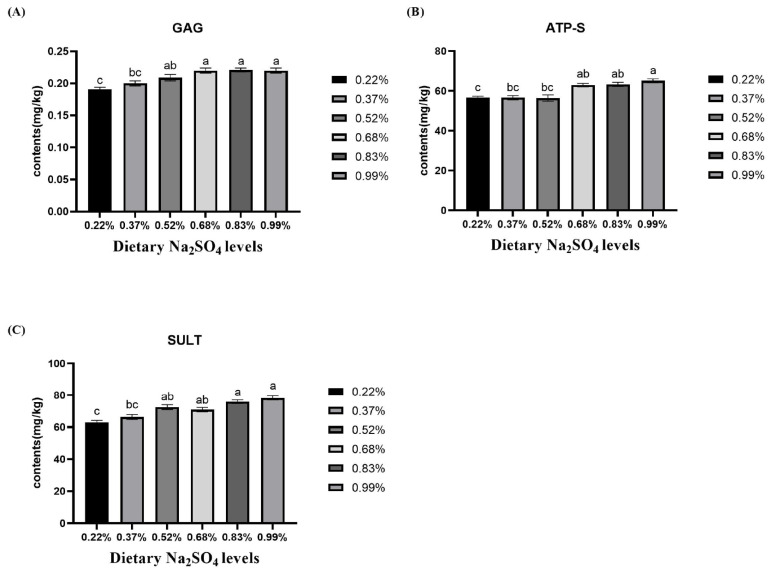
Effect of dietary sodium sulfate (Na_2_SO_4_) supplementation on eggshell sulfated GAG, isthmus ATP-S and SULT contents in the layers. The values are the means of 8 replicates with 4 eggshells each. ^a–c^ The different superscript letters denote significant differences among experimental treatments. GAG, glycosaminoglycan. ATP-S, ATP-sulfurylase; SULT, sulfotransferase.

The organic components of the eggshell increased significantly with increasing dietary Na_2_SO_4_ supplementation (Figure 2). The contents of GAG and ATP-S in the 0.68%, 0.83%, and 0.99% Na_2_SO_4_ groups were greater than those in the 0.22% Na_2_SO_4_ group (p<0.05). Compared with those in the 0.22% and 0.37% Na_2_SO_4_ groups, dramatically greater SULT were observed in the 0.83% and 0.99% Na_2_SO_4_ groups (p<0.05). The CS and DS contents increased dramatically (p<0.05; Table 8).

### Eggshell ultrastructure

Dietary supplementation with 0.68% Na_2_SO_4_ improved eggshell ultrastructure in this study (Supplement 1). We scanned the eggshell ultrastructure electron micrographs of laying hens fed diets containing 0.22%, 0.68%, and 0.99% Na_2_SO_4_. Compared with those of the other groups, the mammillary knob width, mammillary thickness, and percentage of the mammillary layer significantly decreased, whereas the mammillary knob density, effective thickness, and total thickness noticeably increased in the 0.68% Na_2_SO_4_ group (p<0.05; Table 9).

## DISCUSSION

Appropriate supplementation of diets with Na_2_SO_4_ could improve eggshell quality [24]. However, potential risks must be considered, including reductions in laying rate and feed efficiency [4]. Previous research has indicated that there is no significant impact on daily egg weight or laying rate when dietary Na_2_SO_4_ levels range from 0.3% to 0.6% [5]. Likewise, no adverse effects on average egg weight and ADFI were observed when Na_2_SO_4_ was supplemented at levels ranging from 0.22% to 0.99% during the 49 to 60 weeks age group. It is worth mentioning that, although statistically not different, the egg production decreased from 0.37% to 0.99% Na_2_SO_4_ groups compared with dietary 0.22% Na_2_SO_4_ group. It might be that dietary Na levels in these groups exceed the recommended 0.15% [16]. Excessive Na^+^ may be caused the hyperkalemia which lead to laying performance [25], which further resulted in the increment of FCR. Besides, egg production did not decrease with further increased in dietary Na_2_SO_4_ levels. All in all, this result indicated that Na_2_SO_4_ supplementation at 0.22% to 0.99% is safe.

A higher breaking strength of eggshells can reduce economic losses in the layer industry by reducing egg breakage [26]. Na_2_SO_4_ supplementation of 0.52% and 0.68% was found to increase the breaking strength and thickness of laying hens from 49 to 60 wk of age, which was consistent with the findings of previous studies conducted on younger layers (29 to 40 wk of age [7] and 21 to 28 wk of age [4]). These findings indicate that Na_2_SO_4_ is an effective additive for improving eggshell quality. The breaking strength of the eggshell improved notably at a Na_2_SO_4_ supplementation level of 0.68% in our study, which aligns with the recommendation (0.71% Na_2_SO_4_) of Fu et al [7] but is more than the recommended level (0.3% to 0.6% Na_2_SO_4_) reported by Liu et al [4]. This disparity may be due to the differences in the basal dosage of dietary Na and Cl, as the levels and interactions of total Na and Cl in diets should be taken into consideration when supplementing dietary Na [27]. Poor eggshell quality could be caused by higher dietary Na_2_SO_4_ levels, which disturb the balance between Na and Cl, further leading to metabolic alkalosis and changing the secretion of substances necessary for eggshell formation [28]. Overall, the addition of 0.52% and 0.68% dietary Na_2_SO_4_ improved eggshell quality.

CaCO_3_ is the major component of eggshells [29]. In our study, the eggshell weight and eggshell ratio were found to increase with increasing Ca content in the eggshell, suggesting that the increase in thickness may be due to the increase in CaCO_3_ deposition. During eggshell formation, Ca^2+^ transfers from the blood to uterine epithelial cells via the Ca^2+^ channel and then is exchanged with Na^+^ in uterine fluid through *SLC8A1*. Additionally, Na^+^ can also enter uterine epithelial cells through *SCNN1A* and is exchanged with K^+^ in the blood via *ATP1B1*. K^+^ subsequently transported into the uterine fluid via *KCNMA1* [20]. All of them are involved in ion transportation. Ultimately, Ca^2+^ and HCO_3_-combine to form CaCO_3_ in uterine fluid. The transfer of Na^+^ from uterine fluid to serum has a positive relationship with Ca^2+^ transfer from serum to uterine fluid [30]. Therefore, the increased expression of *SLC8A1* and *SCNN1A* led to increased uterine fluid Ca^2+^ levels accompanied by decreased Na^+^ levels in this study. These results indicated that 0.68% Na_2_SO_4_ supplementation could improve the exchange of Ca^2+^ and Na^+^. Additionally, in the 0.68% Na_2_SO_4_ groups, ATP1B1 and KCNMA1 expression increased significantly compared with that in the other groups. The serum Na^+^ concentration and uterine fluid K^+^ concentration increased linearly with Na_2_SO_4_ supplementation, ranging from 0.22% to 0.68%. This finding suggested that the transportation of K^+^ from the serum to the uterine fluid could be improved by dietary 0.68% Na_2_SO_4_ supplementation. Furthermore, the Ca content of the eggshell, ion concentration, and gene expression of ion transporters showed the opposite trend with increasing Na_2_SO_4_ supplementation from 0.68% to 0.99%. These indicated that excessive Na+ decreases the absorption of Ca^2+^ and leads to a decrease in eggshell quality [28]. These two treatments (0.83% and 0.99% Na_2_SO_4_) may exceed their tolerance, which is consistent with the recommendation of Fu et al [31]. The process by which sodium enters the blood occurs against a concentration gradient, while *ATP1B1* consumes energy [32]. An elevated serum Na^+^ concentration may depress the transfer of other ions, and elevated dietary Na_2_SO_4_ levels could disrupt the balance between Na^+^ and Ca^2+^, leading to a decreased Ca^2+^ concentration [4]. Excessive Na^+^ can also reduce the affinity of *SCNN1A* [33], ultimately leading to a weakened ability for subsequent ion exchange. In general, the Na^+^ concentration in the 0.68% group was lower than that in the 0.83% and 0.99% groups, which corresponded to the changes in breaking strength and thickness. Based on these results, the changes in ion concentrations and transporter levels could partly explain the increase in breaking strength in the 0.68% Na_2_SO_4_ group.

Most of the eggshell matrix is considered to be a part of the eggshell structure or a regulator of eggshell mineralization [10]. We measured the eggshell matrix and its associated proteins. Consistent with previous findings [7], our study showed an increase in GAG content with increasing dietary Na_2_SO_4_. Moreover, the contents of CS, DS, ATP-S, and SULT on the isthmus markedly increased with increasing dietary Na_2_SO_4_. There are four types of GAG, namely, heparan sulfate, CS, keratan sulfate (KS), and hyaluronic acid; eggshell GAG contains approximately 70% CS [34–36]. KS is involved in the formation of the first mammillary core, while CS, together with DS and OC-116 (where DS is considered a type of CS), regulates the growth of the palisade layer [36,37]. In addition, SO_4_^2−^ is converted to 3′-phosphoadenosine-5′-phosphosulfate (PAPS) by ATP-S. PAPS is subsequently converted to sulfate GAG by SULT [38]. Crystallization has been reported to occur at calcium, which contains a high concentration of sulfur. Additionally, the effect of GAG on the morphology and size of CaCO_3_ crystals is concentration-dependent [39]. This might also explain why eggshell GAG contents increased. The eggshell CS/DS copolymer was regarded as the major GAG involved in maintaining eggshell strength. It has a particular affinity for Ca^2+^ and can modify the shape of calcite crystals formed by CaCO_3_. This difference may be related to the increase in eggshell quality. However, the 0.83% and 0.99% Na_2_SO_4_ groups did not exhibit a corresponding change in breaking strength. A previous study demonstrated that certain GAG contents did not have any additional effect on eggshells [39]. This difference may account for the difference in eggshell strength and weight between the 0.83% and 0.99% Na_2_SO_4_ groups compared with the 0.68% Na_2_SO_4_ group. Therefore, we suggest that the eggshell GAG contents in the 0.68% Na_2_SO_4_ treatment group could improve eggshell quality and that higher concentrations of GAG would not produce further effects. On the other hand, we speculated that ion concentrations and transporter levels may be the main factors affecting eggshell quality, while GAG content play an auxiliary role in eggshell quality.

The ultrastructure, which consists of the palisade layer, vertical crystal layer, and cuticle layer, is a primary determinant of eggshell quality [40,41]. Alterations in eggshell ultrastructure are crucial factors for improving physical structure, including increasing effective thickness and knob density, as well as total calcification layer thickness and decreasing mammillary thickness [42]. An effective layer strongly affects the breaking strength [43]. In the 0.68% group, both the effective thickness and total calcification layer thickness were significantly greater than those in the other groups. This variation might result from the increased Ca content in the eggshell, which also appears to increase eggshell thickness and subsequently decrease eggshell quality. Wu et al [39] reported that CS/DS proteoglycans from the eggshell membrane could cause the formation of smaller and more rounded CaCO_3_ crystals. An increased mammillary knob density and reduced mammillary knob width and thickness might lead to greater binding between mastoid processes and enhanced resistance to external forces [44]. These changes were consistent with the increase in breaking strength, suggesting that the increase in eggshell quality may be related to the improvement in eggshell ultrastructure.

## CONCLUSION

In conclusion, our results indicate that supplemental 0.22% to 0.99% Na_2_SO_4_ in the corn-soybean basal diet containing 0.15% Cl did not have adverse effects on performance in laying hen. Dietary 0.52% and 0.68% Na_2_SO_4_ supplementation could regulate uterine ion transport and increase GAG contents, which contribute to better eggshell quality. In addition, dietary supplementation with 0.68% Na_2_SO_4_ resulted in higher eggshell breaking strength, thickness, eggshell weight, and eggshell ratio.

## Figures and Tables

**Table 1:** Composition and nutrient content of the basal diet of laying hens (air-dry basis, %)^1)^

Ingredient	Content (%)	Nutrient level	Content (%)
Corn	60.50	AME (kcal/kg)	2,698.96
Soybean meal	18.50	Crude protein	16.54
Extruded soybean	8.50	Methionine	0.37
Limestone	9.02	Lysine	0.83
Calcium phosphate	1.75	Methionine+cystine	0.66
NaCl	0.15	Calcium^2)^	3.62
DL-Methionine (98%)	0.11	Total phosphorus^2)^	0.61
L-Lysine HCl (50%)	0.10	Available phosphorus	0.36
Vitamin and mineral premix^3)^	0.32		
Experimental premix^4)^	1.05		
Total	100		

1) The contents are the calculated values except for the contents of calcium.

2) The values in basal diets were determined by analysis in accordance with the Chinese National Standard [44].

3) Vitamin and mineral premix (provided per kilogram of diet): vitamin A, 12 500 IU; vitamin D_3_, 4 125 IU; vitamin E, 15 IU; vitamin K_3_, 2 mg; riboflavin, 8.5 mg; calcium pantothenate, 50 mg; thiamine, 1 mg; niacin, 32.5 mg; biotin, 2 mg; pyridoxine, 8 mg; folic acid, 5 mg; vitamin B_12_, 5 mg; I, 1 mg; Zn, 66 mg; Fe, 60 mg; Se, 0.3 mg; Cu, 8 mg; Mn, 65 mg.

4) Includes 0.22%, 0.37%, 0.52%, 0.68%, 0.83%, and 0.99% sodium sulfate, respectively. The other parts were supplemented with zeolite powder to 1.05%.

AME, apparent metabolic energy.

**Table 2:** Analyzed sodium and chloride contents in the experimental diets (air-dry basis, %)^1)^

Items^2)^	Dietary sodium sulfate level (%)					

	0.22	0.37	0.52	0.68	0.83	0.99
Total dietary Na level (%)	0.15 (0.16)	0.20 (0.20)	0.25 (0.24)	0.30 (0.29)	0.35 (0.36)	0.40 (0.4^1)^
Total dietary Cl level (%)	0.15 (0.16)	0.15 (0.16)	0.15 (0.16)	0.15 (0.16)	0.15 (0.16)	0.15 (0.16)

1) The numbers in parentheses indicate the analyzed values. The others are calculated values.

2) The values in basal diets were determined by analysis in accordance with the Chinese National Standard [45].

**Table 3:** The primer sequences for the target and reference genes

Gene	Forward/reverse prime	Accession
*SCNN1A* ^1)^	GCTTGCCAGAAAACAGTCCCTC	NM_205145
	AGTCAGACTCATCCAGGTCTTTGG	
*SLC8A1*	GGATTGTGGAGGTTTGGGAAGG	NM_001079473
	CTGTTTGCCAGCTCGGTATTTC	
*ATP1B1*	TCTGGAACTCGGAGAAGAAGGAG	NM_205520
	GACGGTGAGCAACATCACTTGG	
*KCNMA1*	GGGATGATGCGATCTGTCTT	NM_204224
	GACAAACCCACAAAGGCACT	
*β-actin*	TATGTGCAAGGCCGGTTTC	NM_205518
	TGTCTTTCTGGCCCATACCAA	

1) *SCNN1A*, sodium channel epithelial 1 subunit *α*; *SLC8A1*, solute carrier family 8-member a1; *ATP1B1*, ATPase Na^+^/K^+^ transporting subunit *β1*; *KCNMA1*, potassium calcium-activated channel subfamily m α1.

**Table 4:** Effect of dietary sodium sulfate (Na_2_SO_4_) supplementation on performance in laying hens (49 to 60 of age)^1)^

Items	Time (wk)	Dietary sodium sulfate level (%)						SEM	p-value		
	
		0.22	0.37	0.52	0.68	0.83	0.99		ANOVA	Linear	Quadratic
Average egg weight (g)	49–52	65.44	65.08	65.49	65.55	65.04	64.97	0.20	0.931	0.551	0.741
	53–56	64.46	64.39	64.29	64.57	64.12	63.62	0.21	0.830	0.271	0.412
	57–60	64.44	64.43	64.48	64.91	63.78	63.96	0.20	0.670	0.350	0.461
	49–60	64.78	64.63	64.75	65.01	64.32	64.18	0.19	0.852	0.361	0.512
Egg production (%)	49–52	96.28	91.27	93.90	93.25	93.16	94.89	0.55	0.140	0.872	0.183
	53–56	93.55	90.63	91.29	89.78	93.04	90.51	0.54	0.283	0.402	0.421
	57–60	92.36	89.94	91.49	90.99	91.15	91.03	0.53	0.901	0.753	0.842
	49–60	94.06	90.61	92.22	91.34	92.45	92.14	0.42	0.290	0.570	0.304
ADFI^2)^ (g)	49–52	130.78	126.58	128.29	128.85	130.32	131.22	0.58	0.172	0.243	0.081
	53–56	121.72	120.11	120.37	121.13	120.20	119.83	0.45	0.851	0.362	0.662
	57–60	125.26	124.16	124.39	123.29	125.64	124.09	0.44	0.711	0.791	0.801
	49–60	125.92	123.62	124.35	124.42	125.39	125.05	0.31	0.320	0.871	0.302
FCR	49–52	2.15	2.15	2.11	2.13	2.16	2.15	0.01	0.904	0.775	0.691
	53–56	2.06	2.12	2.05	2.16	2.10	2.09	0.02	0.324	0.436	0.500
	57–60	2.12	2.19	2.12	2.10	2.18	2.14	0.01	0.380	0.839	0.964
	49–60	2.11	2.15	2.09	2.13	2.14	2.13	0.01	0.539	0.494	0.792

1) Data represent the mean of 8 replicates (n = 8), each with 9 birds.

SEM, standard error of the mean; ADFI, average daily feed intake; FCR, feed conversion ratio.

**Table 5:** Effect of dietary sodium sulfate (Na_2_SO_4_) supplementation on eggshell quality in laying hens (49 to 60 wk of age)^1)^

Items	Time (wk)	Dietary sodium sulfate level (%)						SEM	p-value		
	
		0.22	0.37	0.52	0.68	0.83	0.99		ANOVA	Linear	Quadratic
Breaking strength (N)	52	45.02	44.20	44.31	44.61	43.17	45.20	0.28	0.371	0.751	0.431
	56	43.54^b^	43.86^b^	45.32^ab^	45.77^a^	43.44^b^	43.42^b^	0.28	0.031	0.810	0.022
	60	41.91^b^	41.20^b^	44.07^a^	44.13^a^	41.91^b^	41.85^b^	0.29	0.004	0.750	0.030
Thickness (mm)	52	0.45	0.46	0.46	0.46	0.45	0.46	<0.01	0.951	0.833	0.941
	56	0.43	0.44	0.44	0.45	0.44	0.43	<0.01	0.052	0.141	0.022
	60	0.44^b^	0.45^ab^	0.45^a^	0.45^a^	0.44^b^	0.44^b^	<0.01	0.002	0.752	<0.001
Eggshell weight (g)	52	6.46	6.21	6.22	6.35	6.07	6.27	0.04	0.061	0.111	0.121
	56	6.21	6.21	6.32	6.39	6.24	6.23	0.02	0.082	0.592	0.071
	60	6.16^b^	6.28^ab^	6.29^ab^	6.39^a^	6.19^b^	6.14^b^	0.02	0.011	0.514	0.003
Eggshell ratio (%)	52	9.99	9.65	9.59	9.82	9.58	9.81	0.05	0.062	0.352	0.072
	56	9.64^b^	9.70^b^	9.90^ab^	10.04^a^	9.85^ab^	9.80^ab^	0.04	0.047	0.091	0.012
	60	9.56^b^	9.77^ab^	9.77^ab^	9.90^a^	9.62^b^	9.56^b^	0.03	0.014	0.720	0.022
Egg shape index	52	1.33	1.34	1.33	1.33	1.32	1.34	<0.01	0.112	0.282	0.520
	56	1.35	1.35	1.35	1.36	1.36	1.35	<0.01	0.640	0.581	0.781
	60	1.36	1.36	1.36	1.35	1.35	1.36	<0.01	0.713	0.203	0.341

1) Data represent the meanof 8 replicates (n = 8), each with 12 eggs.

a,b Means within the same row with no common superscript differ significantly (p<0.05).

SEM, standard error of the mean.

**Table 6:** Effect of dietary sodium sulfate (Na_2_SO_4_) supplementation on the eggshell components of laying hens (60 wk of age)^1)^

Items	Dietary sodium sulfate level (%)						SEM	p-value		
	
	0.22	0.37	0.52	0.68	0.83	0.99		ANOVA	Linear	Quadratic
Ca (%)	32.30^c^	34.28^ab^	34.70^ab^	35.19^a^	34.00^ab^	33.60^b^	0.22	<0.001	0.091	<0.001
P (%)	0.12	0.10	0.11	0.11	0.11	0.11	<0.01	0.122	0.099	0.200
Na (%)	0.12	0.12	0.12	0.12	0.12	0.13	<0.01	0.101	0.026	0.078
S (%)	0.14	0.16	0.15	0.14	0.15	0.15	<0.01	0.187	0.476	0.706

1) Data represent the means of 8 replicates (n = 8), each with 4 eggshells.

a–c Means within the same row with no common superscript differ significantly (p<0.05).

SEM, standard error of the mean; P, phosphorus; S, sulfur.

**Table 7:** Serum and uterine fluid ion concentrations of laying hens at 18.5 h post oviposition (60 wk of age)^1)^

Items	Dietary sodium sulfate level (%)						SEM	p-value		
	
	0.22	0.37	0.52	0.68	0.83	0.99		ANOVA	Linear	Quadratic
Serum										
K^+^ (mmol/L)	4.94	4.89	4.58	4.43	4.18	4.07	0.16	0.571	0.047	0.143
Na^+^ (mmol/L)	152.69^b^	154.34^b^	158.70^ab^	159.05^ab^	164.46^a^	165.05^a^	1.36	0.031	<0.001	0.002
Ca^2+^ (mmol/L)	0.69	0.73	0.79	0.83	0.80	0.81	0.02	0.387	0.049	0.080
Cl^−^ (mmol/L)	107.59	109.38	105.98	104.88	105.76	110.29	0.67	0.122	0.979	0.136
HCO_3_^−^ (mmol/L)	21.98	21.71	23.21	23.49	23.51	22.30	0.41	0.701	0.395	0.365
pH	7.32	7.31	7.32	7.31	7.32	7.26	0.01	0.798	0.333	0.465
Uterine fluid										
K^+^ (mmol/L)	73.69^a^	71.53^ab^	74.73^a^	75.35^a^	61.26^b^	62.12^b^	1.68	0.200	0.008	0.009
Na^+^ (mmol/L)	49.19^ab^	49.10^ab^	44.56^b^	44.53^b^	56.00^a^	56.50^a^	1.61	0.040	0.001	0.006
Ca^2+^ (mmol/L)	6.23^b^	6.41^b^	8.01^ab^	8.80^a^	7.05^b^	6.90^b^	0.26	0.018	0.287	0.011
Cl^−^ (mmol/L)	80.60	79.36	82.03	79.36	81.00	79.47	0.82	0.923	0.848	0.931
HCO_3_^−^ (mmol/L)	46.00	52.10	47.38	50.54	49.68	51.63	1.41	0.809	0.354	0.648
pH	7.51	7.57	7.46	7.41	7.54	7.49	0.02	0.470	0.755	0.672

1) Data represent the mean of 8 replicates (n = 8), each with 2 birds.

a,b Means within the same row with no common superscript differ significantly (p<0.05).

SEM, standard error of the mean.

**Table 8:** Effect of dietary sodium sulfate (Na_2_SO_4_) supplementation on the isthmus chondroitin sulfate and dermatan sulfate contents of laying hens (60 wk of age)^1)^

Items^2)^	Dietary sodium sulfate level (%)						SEM	p-value		
	
	0.22	0.37	0.52	0.68	0.83	0.99		ANOVA	Linear	Quadratic
Chondroitin sulfate (ng/mL)	61.31	56.65	58.79	62.67	66.98	89.56	3.14	0.080	0.021	0.009
Dermatan sulfate (ng/mL)	4.53	4.68	5.49	5.17	7.24	7.56	0.39	0.101	0.005	0.015

1) Data represent the mean of 8 replicates (n = 8), each with 2 birds.

SEM, standard error of the mean; ANOVA, analysis of variance.

**Table 9:** Effect of dietary sodium sulfate (Na_2_SO_4_) supplementation on the eggshell ultrastructure of laying hens (60 wk of age)^1)^

Items	Dietary sodium sulfate level (%)			SEM	ANOVA

	0.22	0.68	0.99		
Mammillary knob width (μm)	92.72^a^	75.11^b^	86.95^a^	2.13	<0.001
Mammillary knob density (1 mm^2^)	278.42^b^	284.56^a^	280.85^ab^	0.92	0.015
Mammillary thickness (μm)	85.63^a^	68.34^b^	78.39^ab^	2.57	0.015
Effective thickness (μm)	269.38^b^	300.98^a^	272.20^b^	3.64	<0.001
Total thickness (μm)	355.00^b^	369.33^a^	350.59^b^	3.12	0.030
Mammillary layer (%)	24.10^a^	18.51^b^	22.27^a^	0.69	<0.001

1) Data represent the mean of 8 replicates (n = 8), each with 4 eggshells.

a,b Means within the same row with no common superscript differ significantly (p<0.05).

SEM, standard error of the mean; ANOVA, analysis of variance.

## SUPPLEMENTARY MATERIAL

**Supplement 1.** Effect of dietary sodium sulfate (Na_2_SO_4_) supplementation on the eggshell ultrastructure of laying hens fed the experimental diets (scanning electron microscope images, magnification, 200×).
