# Supplementary material for: Dietary sodium sulfate supplementation improves eggshell quality, uterine ion transportation and glycosaminoglycan synthesis in laying hens
Source: Anim Biosci. 2024 Oct 28;38(5):1029–40. doi: 10.5713/ab.24.0456 (PMC12062801; doi:10.5713/ab.24.0456)
Supplement: Supplementary file 1 [file ab-24-0456-Supplementary-1.pdf]

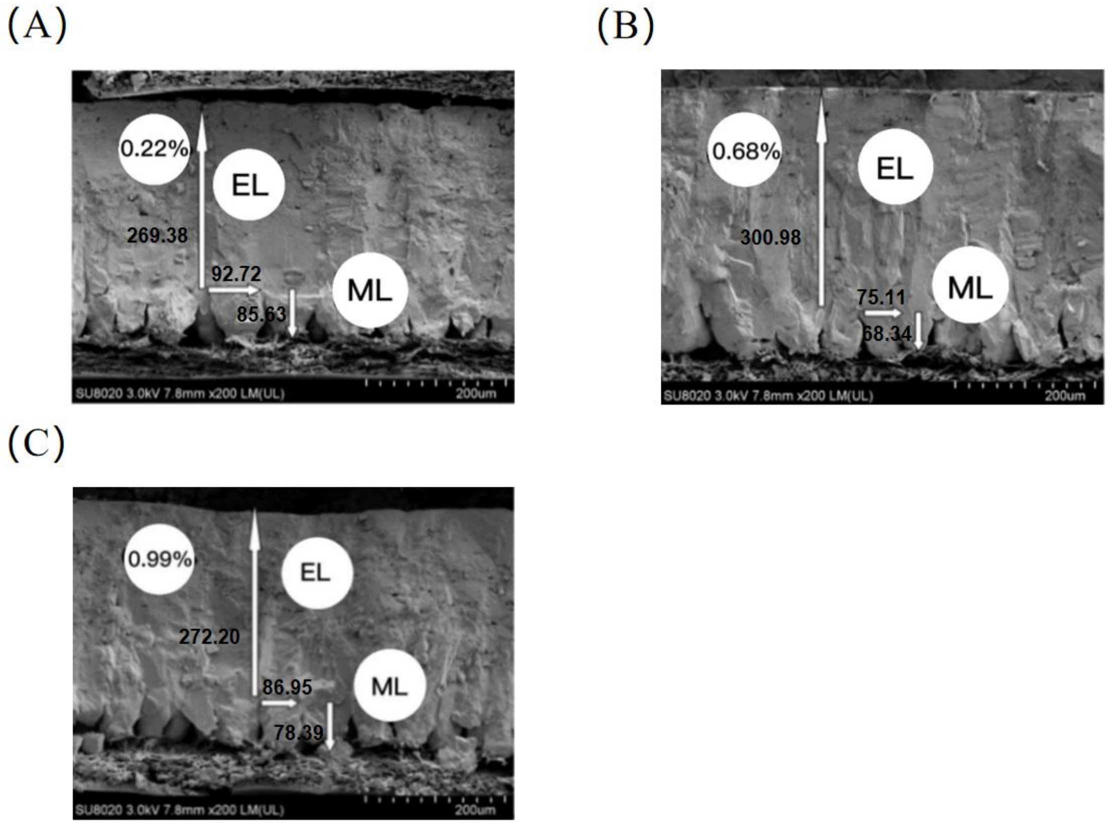

Supplement 1. Effect of dietary sodium sulfate (Na_2SO_4) supplementation on the eggshell ultrastructure of laying hens fed the experimental diets (scanning electron microscope images, magnification, 200 ×). The three treatment groups were fed corn–soybean meal diets supplemented with 0.22%, 0.68%, or 0.99% Na_2SO_4. EL, effective layer; ML, mammillary layer.
